# Remineralization of Initial Carious Lesions Using Peptides: A Comprehensive Review

**DOI:** 10.3390/medicina62061086

**Published:** 2026-06-03

**Authors:** Ruth M. Santamaría, Mohammad Alkilzy, Christian H. Splieth, Julian Schmoeckel

**Affiliations:** Department of Pediatric Dentistry, University Medicine Greifswald, 17475 Greifswald, Germany; alkilzym@uni-greifswald.de (M.A.); splieth@uni-greifswald.de (C.H.S.); julian.schmoeckel@uni-greifswald.de (J.S.)

**Keywords:** enamel remineralization, initial carious lesions, biomimetic remineralisation, self-assembling peptides, P11-4

## Abstract

Initial carious lesions represent a reversible stage of the caries process in which non-operative strategies can prevent lesion progression and preserve dental hard tissues. This comprehensive review provides an overview of peptide-based approaches for the management of initial carious lesions, with emphasis on self-assembling peptides. The literature was identified through PubMed electronic searches complemented by manual screening of reference lists. Only randomized clinical trials and controlled clinical studies published in English were included. The PICOS framework guided the structure of the review, focusing on patients of any age with initial carious lesions, peptide-based interventions aimed at enamel remineralization, comparisons with placebo, alternative treatments, or standard preventive care (e.g., fluoride products), and outcomes related to de-/remineralisation. Overall, the available evidence suggests that peptide-based strategies can mimic natural biomineralization and promote subsurface hydroxyapatite formation. Among the investigated approaches, the self-assembling peptide P11-4 is the most extensively studied. Evidence supports its safety and its potential to enhance initial carious lesion remineralisation, with possible advantages over fluoride alone in selected cases. In conclusion, peptide-based potentially regenerative approaches, particularly P11-4, represent a promising adjunct in minimally invasive caries management, although further long-term and comparative clinical studies are needed to define their role in routine dental practice.

## 1. Introduction

Dental caries remains a major global health challenge and continues to affect a substantial proportion of children, adolescents, and adults worldwide [1,2]. Despite overall declines in prevalence in several industrialised countries, early carious lesions are still frequently detected in clinical practice and represent a critical stage in disease development [3,4,5]. Conventional operative approaches, commonly referred to as “drill and fill,” address the consequences of caries rather than the disease process itself and often initiate a cycle of repeated restorative interventions and progressive loss of sound tooth structure.

Contemporary caries management, therefore, emphasises prevention, early detection, and minimally invasive strategies aimed at preserving dental hard tissues [6,7,8]. Nevertheless, many preventive measures depend heavily on patients’ motivation to implement preventive strategies, which is particularly relevant in children and adolescents. While fluoride-based products and sealants remain the cornerstone of preventive care, their ability to regenerate enamel is limited, particularly in subsurface lesions [9,10,11]. This has led to growing interest in biomimetic approaches that seek not only to arrest lesion progression but also to promote potential enamel regeneration. Biomimetic materials are generally defined as materials designed to mimic the structure, function, or biological processes of natural tissues or systems in order to potentially support repair or regeneration [12]. In dentistry, peptide-based technologies have emerged as a promising strategy to restore enamel-like mineral structures in early carious lesions.

### Rationale for Peptide-Based Regeneration

Dental enamel is a highly mineralised tissue that is mainly composed of hydroxyapatite crystals organised in a prismatic structure. This structure gives enamel its hardness and resistance to mechanical forces. However, as enamel lacks cellular components, it has no intrinsic capacity for biological repair once mineral loss has occurred. Initial carious lesions develop when the balance between demineralisation and remineralisation is disturbed. The metabolism of fermentable carbohydrates by plaque bacteria produces organic acids that lower the pH at the tooth surface, leading to the dissolution of minerals from enamel. In the early stages, mineral loss occurs beneath an apparently intact enamel surface layer, resulting in a subsurface porous lesion. This occurs because the superficial enamel layer may remain relatively preserved due to partial remineralization from saliva, while ongoing acid diffusion causes continued mineral loss in the underlying enamel. At this stage, the process is potentially reversible if remineralisation exceeds ongoing demineralisation [13,14]. Remineralisation depends on the availability of essential minerals, such as calcium and phosphate ions, as well as an adequate pH environment and buffer factors, such as saliva. Saliva plays a key role in this process by buffering acids, supplying minerals and facilitating the re-precipitation of minerals into partially demineralised enamel [15]. Fluoride enhances this process by promoting the formation of more acid-resistant mineral phases and reducing further mineral loss. However, conventional fluoride-based approaches primarily act at or near the lesion surface, which may limit deeper mineral penetration into the subsurface lesion [13].

True regeneration of initial carious lesions requires remineralisation to occur not only at the enamel surface, but also within the lesion itself. This requires remineralising agents to penetrate the lesion and promote the formation of minerals that closely resemble the composition and structural organisation of natural enamel. While conventional preventive agents primarily enhance surface remineralisation and increase resistance to acid challenges, they often fail to restore the enamel’s original structural organisation throughout the lesion.

**Figure 1:**
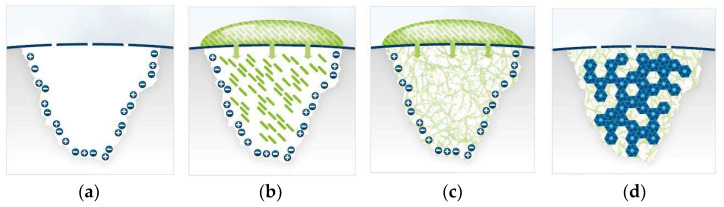
(**a**–**d**) Schematic illustration of the proposed mechanism of biomimetic enamel remineralisation by self-assembling peptide P11-4 in initial carious lesions. Presence of an initial carious lesion (**a**); A drop of Curodont™ Repair (green) is applied to the tooth surface after etching with phosphoric acid and diffuses through the pores of the surface layer into the carious lesion (**b**); within the lesion, Curodont™ Repair forms a three-dimensional network (**c**). Over a period of 1–6 months, new hydroxyapatite crystals form around the Curodont™ Repair network, resulting in potential “regeneration” or remineralisation of the carious lesion (**d**). (Graphic formerly provided by Credentis AG, reproduced with permission).

Consequently, regenerative strategies aim to support the guided, localized deposition of minerals within the lesion, enabling completer and more stable repair of early enamel lesions. In this context, different classes of peptides have been investigated (e.g., self-assembling peptides, amelogenin-derived peptides, and synthetic and engineered peptides) for their ability to promote enamel regeneration by mimicking natural biomineralisation processes. Despite their differences in origin, molecular structure and mechanisms of action, these peptides share the common goal of facilitating hydroxyapatite formation and subsurface lesion remineralization at the molecular level [16,17,18,19]. Among these, the self-assembling peptide P11-4 has been extensively investigated as a biomimetic approach for the management of initial carious lesions. P11-4 is designed to diffuse into the porous subsurface lesion, where it self-assembles in situ into a three-dimensional biomimetic scaffold that facilitates hydroxyapatite nucleation and promotes subsurface remineralisation (Figure 1). By harnessing natural salivary mineralisation processes, this approach aims to support lesion repair in a minimally invasive and biologically driven manner. Numerous in vitro and in vivo studies have analysed the potential of P11-4 for biomimetic remineralization and enamel regeneration, supporting its role as an innovative treatment option for the management of early-stage carious lesions [18,19,20,21].

## 2. Literature Search and Methodological Approach

- This work provides a comprehensive review with a narrative character, informed by a systematic literature search in PubMed, aiming to deliver a structured and evidence-based overview of the topic. The PICOS framework was used to guide study eligibility, search strategy, and content organisation: Population (P): patients of any age presenting with initial carious lesions;
- Intervention (I): peptide-based therapies aimed at enamel regeneration;
- Comparison (C): placebo, no treatment, another treatment (e.g., resin infiltration), or standard care (e.g., fluoride-based products such as varnishes or gels);
- Outcomes (O): outcomes related to enamel demineralisation and/or remineralisation, independent of the assessment method used (e.g., visual scoring systems, laser fluorescence, etc.);
- Study design (S): randomised controlled trials (RCTs) and prospective controlled clinical trials (CCTs).

The literature search was conducted primarily in PubMed/MEDLINE, to identify relevant English-language studies published between 2000 and 3 January 2026. Search terms were developed based on the PICOS framework and adapted to the objectives and narrative character of this review.

**Figure 2:**
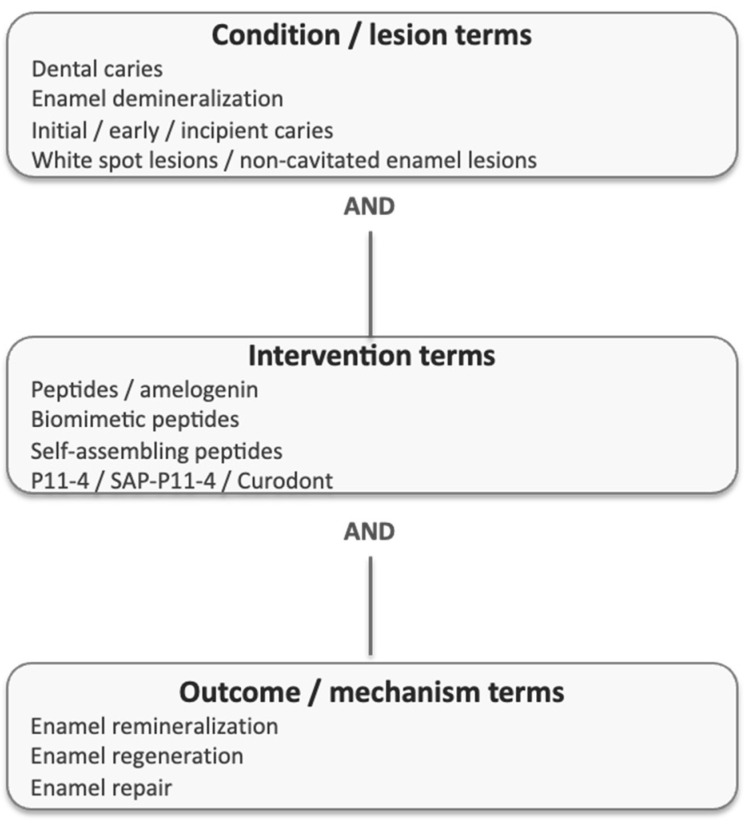
Search strategy diagram and key search terms used for the comprehensive narrative review.

Titles and abstracts were screened for relevance, followed by full-text assessment of potentially eligible studies (Figure 2). The literature search on 3 January 2026 was guided by representative keywords and MeSH terms, combining terms related to dental caries and enamel demineralization (“Dental Caries” [MeSH] OR “Enamel Demineralization” [MeSH] OR initial caries OR early caries OR incipient caries OR white spot lesions OR enamel lesions), peptide-based interventions (“Peptides” [MeSH] OR “Amelogenin” [MeSH] OR peptide OR biomimetic peptide OR self-assembling peptide* OR P11-4 OR enamel regeneration peptide*), enamel remineralization/regeneration (“Enamel Remineralization” [MeSH] OR “Tooth Remineralization” [MeSH] OR remineralization OR remineralisation OR demineralization OR demineralisation OR enamel regeneration OR enamel repair*), and conventional preventive care comparators (placebo* OR fluoride* OR “standard care” OR “preventive care”). Only clinical studies meeting the predefined PICOS criteria were included. On the other hand, laboratory studies, animal studies, observational cohort studies, case reports, and retrospective analyses were excluded. Given the heterogeneity of study designs, outcome measures, and follow-up periods, a qualitative synthesis was performed. A formal risk-of-bias assessment was not undertaken, as the primary aim of this comprehensive narrative review was to provide a structured overview of existing clinical evidence and identify current knowledge gaps, rather than to perform a formal systematic evidence synthesis or generate pooled effect estimates through meta-analysis.

## 3. Results

All eligible studies were independently reviewed in full by an experienced investigator for data extraction. The accuracy of the extracted data was subsequently verified by a second senior researcher. The main findings of the included studies are summarized below.

### 3.1. Types of Peptides Used for Enamel Regeneration

Different classes of peptides have been investigated for their potential to promote enamel regeneration by mimicking natural biomineralisation processes all aim to support hydroxyapatite formation and lesion remineralisation at the molecular level (Table 1).

#### 3.1.1. Self-Assembling Peptides

Self-assembling peptides are the most extensively studied peptide group for enamel regeneration, with evidence from in vitro, in situ, and clinical studies supporting its remineralisation potential. Its application aims to promote natural enamel regeneration by mimicking endogenous remineralisation processes [17,19,20,21,27].

#### 3.1.2. Amelogenin-Derived Peptides

Amelogenin-derived peptides are based on fragments of natural enamel matrix proteins involved in physiological enamel formation during tooth development. These peptides aim to replicate specific functional domains of amelogenin that regulate crystal growth and orientation. Experimental studies have demonstrated their ability to guide enamel-like crystal formation; however, most investigations remain at the in vitro or early preclinical stage, and clinical evidence is currently limited [28].

#### 3.1.3. Synthetic and Engineered Peptides

Synthetic and engineered peptides are designed to optimise stability, binding affinity, and remineralisation capacity. These peptides are often modified to enhance interaction with enamel surfaces or mineral ions. While promising results have been reported in laboratory models, their translation into clinical application is still under development, and long-term clinical data are lacking.

### 3.2. Self-Assembling Peptides: Peptide P11-4

#### 3.2.1. Mechanisms of Action

Peptide P11-4 is a self-assembling peptide composed of 11 amino acids (Ace-Gln-Gln-Arg-Phe-Glu-Trp-Glu-Phe-Glu-Gln-Gln-NH_2_). P11-4 can infiltrate subsurface enamel lesions and spontaneously assemble into three-dimensional scaffolds that act as nucleation templates for hydroxyapatite formation. Their ability to form organised matrices within demineralised enamel makes them particularly suitable for the treatment of initial, non-cavitated carious lesions. In this type of lesions P11-4 penetrates the subsurface enamel, where the peptides self-assemble into elongated structures resembling ladder rungs. This peptide scaffold acts as a matrix that attracts and incorporates calcium, phosphate, and hydroxyl ions, thereby promoting hydroxyapatite formation. The peptide’s high affinity for hydroxyapatite enhances this process, and the addition of fluoride further supports enamel remineralisation [12,20].

#### 3.2.2. Safety and Biocompatibility

Experimental studies have shown that the self-assembling peptide P11-4 does not exhibit cytotoxic properties or trigger immunological responses [18,20]. These findings are supported by in vivo investigations and retrospective clinical studies, which consistently report a favourable safety profile for P11-4. No treatment-related adverse events, medical complications or allergic reactions were observed across these studies, either immediately after application or during follow-up periods of up to three and six months [27]. The clinical safety of P11-4 has been further substantiated by a recent systematic review including meta-analyses, which confirm its suitability for use in patients and reinforce its biocompatibility and clinical applicability [20]. Table 2 provides an overview of the randomized controlled trials (RCTs) included in this review and summarizes their main characteristics and principal findings.

#### 3.2.3. New Evidence

Over the past decade, a growing body of research has examined the clinical effectiveness of the self-assembling peptide P11-4 in managing initial carious lesions. This evidence comprises well-designed randomized controlled clinical trials, clinical studies (Table 2), and in vitro investigations. The information displayed in Table 2 is ordered by year of publication and does not reflect study quality or reported outcomes.

Around 2019, a modified formulation of the self-assembling peptide P11-4, which incorporates 500 ppm sodium fluoride, was introduced to clinical use. A recent clinical investigation compared the remineralization efficacy of this peptide–fluoride formulation with that of fluoride varnish alone (NaF, Bifluorid 10) in 58 early carious lesions among 28 patients [36]. Lesion changes were monitored using laser fluorescence measurements (DIAGNOdent) at baseline as well as after 1, 3, and 6 months.

While both treatments resulted in improvements over time, the peptide–fluoride group exhibited significantly greater remineralization at the 3- and 6-month evaluations (*p* < 0.05; Table 2). These results indicate that the adjunctive use of P11-4 with fluoride may enhance the management of early carious lesions beyond what can be achieved with high-concentration fluoride varnish alone. At present, direct comparative studies between P11-4 alone and Curodont™ Repair Fluoride Plus are lacking. Such investigations would be valuable in clarifying the specific contribution of fluoride within peptide-based remineralisation approaches and in refining the assessment of their clinical effectiveness. In general, all studies consistently support the remineralization potential and clinical applicability of P11-4.

## 4. Discussion

Although a growing number of studies have investigated the self-assembling peptide P11-4, the overall volume of evidence remains limited when compared with other established approaches in minimal and non-invasive caries management for early lesions. Several systematic reviews and meta-analyses have pointed out methodological limitations within the current literature, including small study populations, heterogeneity in outcome measures, and relatively short observation periods [12,20]. Importantly, however, the restricted quantity of available studies should not be interpreted as evidence of ineffectiveness. On the contrary, published investigations to date consistently report favourable safety profiles and positive effects of P11-4 on enamel remineralization across different study designs [12,20,21,22], with associated risks considered minimal due to its application to acellular enamel and its degradation into natural amino acids or excretion if ingested [17]. Although experimental and in situ studies have shown potential of other peptide-based approaches than P11-4, these remain in the research and developmental stage and are not yet widely available for routine clinical use.

Results from clinical and experimental studies suggest that P11-4 may achieve higher levels of caries arrest and remineralization than conventional preventive measures such as sodium fluoride varnish. Nonetheless, head-to-head comparisons with other minimally invasive treatment options are rare. Only a single study has reported superior outcomes for resin infiltration compared with P11-4 [29]. As a result, it remains uncertain whether P11-4 offers should be favored regarding remineralization over alternative non-invasive or minimally invasive strategies, including resin infiltration or silver diamine fluoride (SDF), and further comparative research is warranted.

Based on the currently available evidence, P11-4 can be considered a complementary option for the management of initial carious lesions on various tooth surfaces, including occlusal, approximal, and buccal sites. Its use may be particularly appropriate in clinical situations where fluoride-based measures alone do not achieve sufficient lesion control, while operative intervention is not yet justified [12,22]. In such cases, factors such as patient cooperation, treatment acceptance, and personal economic considerations and structure of the dental healthcare coverage may strongly influence clinical decision-making.

Furthermore, as a relatively recent innovation, the clinical technique and underlying concept of P11-4 remain unfamiliar to many practitioners. Limited exposure and training may reduce confidence in its application and lead to its omission during patient counselling. Similarly to resin infiltration, peptide application does not result in an immediately visible change for the patient, which may complicate communication and acceptance of the treatment. In addition, evaluating treatment success in daily practice remains challenging. Objective assessment tools such as laser fluorescence devices are mainly confined to research settings and are not routinely available in general dental practices. Conventional radiographic imaging also fails to visualise changes associated with peptide-induced remineralisation, making longitudinal monitoring difficult for both clinicians and patients.

This review brings together the currently available evidence supporting the remineralization potential of self-assembling peptides (P11-4), drawing on findings from in vitro, in situ, and clinical studies. However, several limitations inherent to the narrative design of this review should be acknowledged. Although informed by a structured literature search in PubMed, the search was not intended to be fully exhaustive across multiple databases, as would be expected in a formal systematic review. Consequently, some relevant studies may not have been identified. Moreso, no formal risk-of-bias assessment was conducted, due to the nature of this review, which presents another methodological limitation. Nevertheless, the approach in this work provides a clinically focused and structured overview of the currently available evidence while highlighting important areas requiring further research.

Beyond caries management, emerging evidence indicates that P11-4 may have broader applications. Recent studies suggest potential protective effects against enamel erosion, indicating a possible role in preventive strategies beyond caries control [38]. These findings open perspectives for the use of P11-4 in conditions such as erosive tooth wear and dentin hypersensitivity. However, robust clinical studies are still needed to confirm its effectiveness in these indications and to compare it with established treatment approaches.

## 5. Conclusions

In conclusion, P11-4 represents an innovative, biologically driven, and minimally invasive addition to modern caries management. Unlike traditional restorative interventions based on tissue removal, this peptide-based approach aims to promote natural enamel repair and preserve tooth structure. From a practical clinical perspective, P11-4 appears to be a user-friendly and feasible option for the remineralization of initial carious lesions, offering clinicians an additional non-invasive treatment approach that may be readily integrated into preventive and minimally invasive care strategies. However, further well-designed long-term clinical studies are essential to clarify its comparative effectiveness and to possibly support broader integration into routine dental care.

## Figures and Tables

**Table 1:** Comparison of peptide-based approaches for enamel regeneration.

Peptide Type	Concept	Mechanism of Action	Evidence Level	Clinical Availability
Self-assembling peptides (e.g., P11-4) [16,19,22]	Synthetic biomimetic peptides	Infiltrate subsurface lesions and self-assemble into 3D scaffolds promoting hydroxyapatite nucleation	In vitro, in situ, clinical studies	Clinically available
Amelogenin-derived peptides [23,24,25]	Fragments of natural enamel matrix proteins	Mimic physiological enamel formation by guiding crystal orientation and growth	In vitro	Not clinically available
Synthetic/engineered peptides [25,26]	Rationally designed peptide sequences	Enhanced mineral binding and ion attraction to promote remineralisation	In vitro	Not clinically available

**Table 2:** In vivo/clinical studies on the self-assembling peptide P11-4.

Authors (Year)	Methodology	Sample Size	Main Findings
Alkilzy et al. 2018 [27]	RCT comparing peptide P11-4 combined with fluoride varnish (TG) versus fluoride varnish alone (CG) in children with active initial occlusal caries on erupting first and second permanent molars. Outcomes were assessed using LF and visual examination (ICDAS and Nyvad criteria).	62 children, 70 teeth (TG n = 35; CG n = 35)	The TG showed significant improvements at 3 and 6 months. Compared with the CG, lower LF values were observed (OR = 3.5, *p* = 0.015), higher rates of caries regression according to ICDAS (OR = 5.1, *p* = 0.018), and a greater conversion of active lesions to inactive lesions based on Nyvad criteria (OR = 12.2, *p* < 0.0001).
Gözetici et al. 2019 [29]	Split-mouth study comparing three intervention for the treatment of initial caries lesions. LF measurements were taken at 1 week, 3, and 6 months.	21 patients, 84 lesions (21/group); Resin infiltration vs. P11-4 vs. fluoride varnish vs. oral hygiene instruction (CG)	All treatment modalities, including the CG, showed significant reductions in LF values after 6 months. Resin infiltration achieved the greatest lesion regression, followed by fluoride varnish, peptide P11-4, and the control.
Bröseler et al. 2020 [30]	Prospective, randomised split-mouth study comparing peptide P11-4 with fluoride varnish for the treatment of initial buccal carious lesions. Lesion size was evaluated using standardised photographs.	44 patients, 88 lesions (TG n = 44; CG n = 44)	P11-4 resulted in a significant reduction in lesion size, while control lesions remained stable (*p* = 0.001).
Doberdoli et al. 2020 [31]	RCT investigating the treatment of initial occlusal carious lesions in children and adolescents: - TG 1: P11-4 (day 0) + fluoride varnish (baseline and day 180) - TG 2: P11-4 (baseline) + peptide P11-4 applied twice weekly at home - CG: fluoride varnish (baseline and day 180) Caries progression was evaluated after one year using LF, Nyvad caries activity criteria, and ICDAS scores.	90 participants, 90 lesions (30/group); TG1: P11-4 + fluoride varnish; TG2: P11-4 + home peptide application; CG: fluoride varnish	P11-4 led to a significant reduction in occlusal lesion size over the study period. Both test groups showed significant lesion regression, while control lesions progressed (*p* < 0.0005). According to ICDAS, regression was observed in test group 1 (6.7%) and test group 2 (20.0%), whereas progression occurred in the control group (23.3%) (*p* < 0.01). Nyvad scores confirmed greater caries inactivation in the test groups (*p* = 0.002).
Welk et al. 2020 [32]	Split-mouth RCT evaluating peptide P11-4 compared with no treatment for initial smooth-surface caries following orthodontic treatment. Test teeth received the peptide P11-4 at baseline, while control teeth remained untreated. Primary endpoint: lesion assessment using electrical impedance spectroscopy with an individually customised splint. Secondary endpoint: morphometric reduction in lesion size.	23 patients, 46 lesions (TG n = 23; CG n = 23)	A significant reduction in lesion progression was observed with P11-4 compared with no treatment. Impedance reduction was greater in the TG (57.8%) than in the CG (19.8%), and lesion size reduction was also greater in the TG (26.1%) compared with CG (16.2%).
Atteya et al. 2023 [33]	RCT including young patients with initial carious lesions (ICDAS 1-2) on buccal surfaces of permanent teeth. A total of 147 teeth were randomly allocated to three groups: self-assembling peptide P11-4, nanosilver fluoride (NSF), or sodium fluoride (NaF). Lesions were assessed at baseline and after 1, 3, 6, and 12 months using ICDAS scores, lesion activity according to Nyvad criteria, and LF readings.	50 patients, 147 teeth (21/group); P11-4 vs. nano-silver fluoride vs. sodium fluoride	All groups demonstrated progressive improvement in ICDAS scores, lesion activity, and LF readings over time. Significant differences in ICDAS score changes among groups were observed at 3 and 6 months (*p* = 0.005). After 12 months, the greatest reduction in ICDAS scores was observed in the P11-4 group (54.5%), which also showed the highest proportion of inactive lesions according to Nyvad criteria. However, multilevel logistic regression revealed no statistically significant difference in ICDAS score reduction between P11-4 or NSF compared with NaF (AOR = 2.56 and 2.12, respectively
Natchiyar et al. 2023 [34]	RCT including children aged 3–5 years presenting with ICDAS 1-2 in primary anterior teeth. A total of 60 teeth were randomly allocated to receive either self-assembling peptide P11-4 (Curodont™ Repair) or fluoride varnish with xylitol-coated calcium phosphate (Embrace™ Varnish). Lesions were assessed at baseline and after 6 months using ICDAS scores and morphometric analysis of lesion area. Enamel permeability was evaluated as a secondary outcome using scanning electron microscopy of polyvinyl siloxane impressions.	30 children, 60 teeth (TG n = 30; CG n = 30)	The P11-4 group showed a statistically significant reduction in ICDAS scores (*p* = 0.05) and lesion area on morphometric analysis (*p* = 0.008) after 6 months, whereas no significant improvement was observed in the control group. SEM analysis revealed no significant changes in enamel permeability in either group. No statistically significant differences were detected between P11-4 and control varnish across the evaluated outcomes.
Gohar et al. 2023 [35]	RCT including participants presenting with post-orthodontic ICDAS scores 1-2 lesions. Participants were randomly allocated to receive either a fluoride varnish containing 22,600 ppm fluoride with tricalcium phosphate or a biomimetic self-assembling peptide. Lesions were assessed at baseline, 3 months, and 6 months using LF measurements and ICDAS visual scoring.	44 patients, 88 lesions (TG n = 44; CG n = 44)	LF readings showed a statistically significant difference between groups, with lower fluorescence values observed in the self-assembling peptide group, indicating greater subsurface remineralization compared with the fluoride-based varnish. Both groups demonstrated significant improvement over time (*p* < 0.001). Visual assessment using ICDAS scores revealed no significant differences between groups at any follow-up point, suggesting comparable clinical masking of white spot lesions.
Shaalan et al. 2024 [36]	RCT including participants presenting with non-cavitated ICDAS 1-2 carious lesions. Participants were randomly allocated to receive either self-assembling peptide P11-4 combined with fluoride (Curodont Repair Fluoride Plus™) or sodium fluoride varnish (Bifluorid 10). Lesions were assessed at baseline and after 1, 3, and 6 months by two calibrated, blinded examiners using LF.	29 patients, 58 lesions (TG n = 29; CG n = 29)	Both groups showed significant improvement in LF scores over time (*p* < 0.05). No significant difference between groups was observed at 1 month; however, at 3 and 6 months, significantly lower LF readings were detected in the P11-4 + fluoride group compared with fluoride varnish alone (*p* < 0.05). After 6 months, the P11-4 + fluoride group showed a 60% lower risk of caries progression and converted 65.5% of lesions from DIAGNOdent score 3 to score 1, whereas fluoride varnish alone achieved this conversion in 13.8% of lesions.
Bardis Salah Abd Elaziz et al. 2025 [37]	RCT, triple-blind trial including children aged 8–12 years with moderate caries risk (CAMBRA) and visible ICDAS 1-2 lesions on the labial surfaces of maxillary permanent anterior teeth. A total of 39 ICDAS 1-2 lesions were randomly allocated to three groups (n = 13/group): Color change (ΔE) was assessed using a spectrophotometer, and lesion dimensions were evaluated by digital photography at baseline and after 3, 6, and 9 months. Remineralizing agents were applied at baseline, 3, and 6 months.	39 children, 39 lesions (13/group); P11-4 vs. 2% arginine-enriched sodium fluoride vs. functionalised tricalcium phosphate fluoride varnish	All groups showed progressive improvement in lesions color and size over time. At 9 months, arginine-enriched sodium fluoride and functionalized tricalcium phosphate fluoride demonstrated significantly greater color improvement than P11-4 (ΔE = 9.37 ± 3.79 and 9.15 ± 2.74 vs. 12.21 ± 3.03, respectively). Arginine-enriched sodium fluoride achieved the greatest reduction in lesion dimensions, outperforming both P11-4 and tricalcium phosphate fluoride, while P11-4 showed the least clinical improvement overall.

AOR: Adjusted odds ratio, CAMBRA: Caries management by risk assessment, CG: Control group, ΔE: Color difference, LF: DIAGNOdent laser fluorescence, ICDAS: International Caries Detection and Assessment System, NaF: Sodium fluoride, NSF: Nanosilver fluorid, OR: Odds ratio, ppm: Parts per million, RCT: Randomized controlled trial, SEM: Scanning electron microscopy, TG: Test group.

## Data Availability

The original contributions presented in this study are included in the article. Further inquiries can be directed to the corresponding author.
